# FRCP-YOLO: Road object detection algorithm based on improved YOLOv8n

**DOI:** 10.1371/journal.pone.0342084

**Published:** 2026-02-20

**Authors:** Dongmei Liu, Changchun Wang, Xuejun Li, Xiguo Zhao, Chuanli Yin, Yuchi Liu, Shuai Li, Xuan Li

**Affiliations:** 1 School of Electronic Information Engineering, Changchun University, Changchun, Jilin, China; 2 Changchun Tongshi Optoelectronics Technology Co., Ltd., Changchun, Jilin, China; CCET: Chandigarh College of Engineering and Technology, INDIA

## Abstract

The accuracy of road object detection is crucial for ensuring the safe driving of autonomous vehicles. Challenges such as small object missed detection, excessive parameters, low accuracy, and poor robustness are commonly observed in current road object detection models. To address the above problems, a road object detection model named FRCP-YOLO is proposed in the present study, which is developed based on the YOLOv8n. Firstly, to reduce the model’s parameters and complexity, the C2f module in the backbone network is replaced with a lightweight FasterNet Block, which enhancing the speed of image feature extraction; then the proposed R-CA module, which is based on a residual block with the Coordinate Attention (CA) mechanism, is introduced to enhance the model’s focus on objects of interest and improve its feature-learning capability. Secondly, to enhance small object detection performance, a high-resolution branch for feature extraction and a detection head for processing these features are introduced, thereby improving the model’s robustness. Finally, PIoU v2 is selected as the bounding box regression loss function to effectively prevent anchor box enlargement, enhance the ability to focus on anchor boxes, and further improve overall detection accuracy. Based on the KITTI dataset, the comparison experiments between FRCP-YOLO and other mainstream algorithms were carried out, FRCP-YOLO achieves object detection accuracies of 0.924 and 0.667 (in terms of mAP@50 and mAP@50–95) on the test set, representing improvements of 5.0% and 6.6% over the baseline model, while reducing parameters by 4%. Comparative experiments were conducted on the BDD100K dataset of complex road scenes. The detection accuracy of FRCP-YOLO outperforms other mainstream algorithms in challenging scenarios, such as dense traffic, occlusions, and night conditions, which verifies the generalization of FRCP-YOLO, highlighting its reliability and effective object detection capabilities in complex scenarios.

## 1 Introduction

As autonomous driving technology advances rapidly [1–3], research on road object detection, which perceives the surrounding environment [4,5], traffic conditions [6,7], and supports accurate driving decisions, has gained prominence. Road object detection aims to accurately perceive the external environment, especially to accurately identify and locate various objects in complex road environments such as dense occlusion, night and rainy days [8]. By detecting vehicles [9,10], pedestrians [11,12], lane lines [13,14], etc. on the road, potential dangers can be discovered in time, thereby achieving early warning, reducing the risk of traffic accidents [15], and improving the driving experience. As a core component of the autonomous driving system, the detection accuracy of road object detection technology is crucial for ensuring vehicle safety. Accurate road object detection can build a more intelligent and efficient traffic management system [16], and has significant practical value in improving road capacity and safety, optimizing traffic flow management, and improving the level of intelligent driving.

Road object detection algorithm based on deep learning has attracted much attention from researchers for its accuracy and efficiency. Currently, it is mainly exploring and improving multiple directions such as loss function, small object feature extraction method, network architecture, etc., and is committed to further improving the performance of object detection. Yang et al. [17] embedded a deformable convolution network (DCN) in the C2f module of the Backbone network to improve the model’s feature extraction ability under complex background conditions. And they added a global attention mechanism (GAM) to the Neck network to highlight important feature information. Wang et al. [18] embedded the BiFormer attention mechanism in the Neck layer of the original YOLOv8n model to effectively capture the association and dependency between features. Meanwhile, WIoU v3 was used as the loss function to enhance the model’s focus on high-quality anchor boxes. Dang et al. [19] added an attention module to the YOLOv5 backbone network to suppress non-critical information and used CIoU as the loss function to improve vehicle detection accuracy. Chen et al. [20] improved the detection accuracy of small objects by adding a small object detection head to the YOLOX network, and used the deep separable convolution method to optimize the Neck network, further improving the model’s computational efficiency. LI et al. [21] introduced spatial deep convolution (SPD-Conv) to replace the original convolution layer to solve the problem of small object detection in the network; in order to make the network pay more attention to the key information in the feature map, the NAM module was added after the C3 module in the backbone network. Liu et al. [22] proposed a high-resolution detection network (HRDNet) for detecting small objects, in which high-resolution input will be sent to a shallow network to retain more position information, and low-resolution input will be sent to a deep network to extract more semantics. Liu et al. [23] proposed a feature sharing detection head to reduce the redundant parameters of the model; the DWR module was used to enhance the feature extraction capability of the backbone network. Wang et al. [24] proposed an improved feature pyramid network AF-FPN, which uses an adaptive attention module (AAM) and a feature enhancement module (FEM) to reduce information loss in the feature map generation process and enhance the representation capability of the feature pyramid. Zhang et al. [25] reconstructed the YOLOv7 backbone network using the Res3Unit structure to improve the model’s ability to obtain more nonlinear features, and added a mixed attention mechanism module ACmix after the SPPCSPC layer to enhance the model’s attention to vehicles. The above improved algorithms have achieved better results in the road object detection task, but there is still room for improvement in the detection of small objects, the number of model parameters, and the detection accuracy, while the robustness in complex traffic scenarios needs to be improved.

The main contributions of the present study are as follows:

1) To effectively reduce model’s parameters and computational complexity while improving the computational efficiency, the lightweight and fast FasterNet Block is utilized for road object feature extraction. Furthermore, to effectively overcome the model gradient disappearance and performance degradation problems, enhance the detection effect of the object of interest, and improve the detection accuracy, an efficient R-CA module is proposed by combining the residual block in the residual network with the CA mechanism.
2) To enhance small object detection and mitigate missed detections, the small object feature extraction network within the Neck network is improved. Specifically, a dedicated branch for capturing small object feature is incorporated. Additionally, a specialized detection head for small object feature processing is integrated into the Head network, thereby boosting model performance in complex road scenes and overall detection accuracy.
3) PIoU v2 is used as the bounding box regression loss function, which enhances the bounding box regression speed and improves the road object positioning accuracy of the algorithm. Incorporating a penalty factor into PIoU v2 effectively prevents anchor boxes from becoming excessively large, while the introduction of a non-monotonic attention function further improves focus on anchor boxes.

## 2 Related work

### 2.1 YOLOv8n

**Fig 1:**
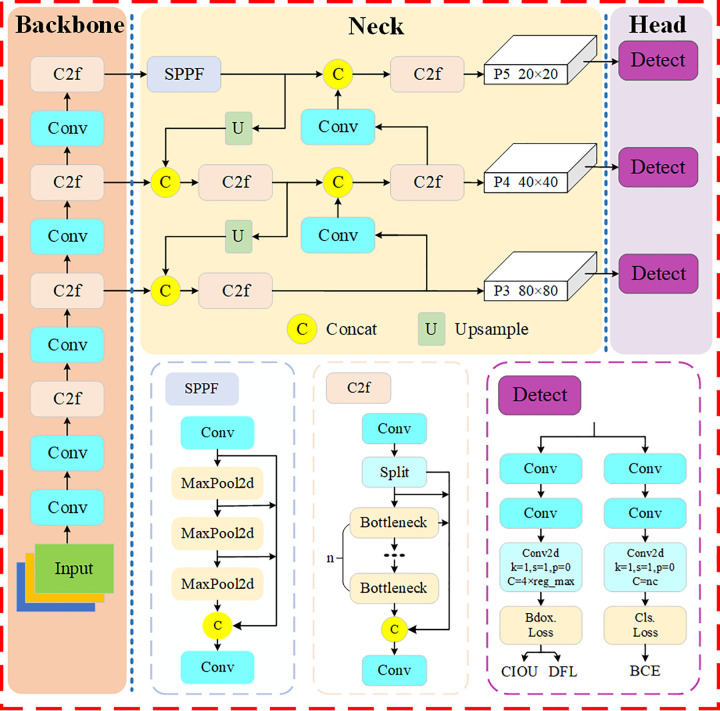
YOLOv8n model structure diagram.

YOLOv8 [26] achieves precise positioning and detection of objects by optimizing network architecture, lightweight design, and utilizing multiscale feature fusion. It is well-suited for autonomous driving applications scenarios that demand real-time performance and high accuracy, and it is straightforward to use and deploy. According to the model’s parameters and performance, YOLOv8 is divided into five versions, among which YOLOv8n is the lightest and fastest network. Due to the limited computing resources of autonomous driving on-board equipment, the object detection model used is required to be both lightweight and fast, YOLOv8n perfectly meets this requirement; it has achieved excellent detection accuracy on public datasets and is suitable for detecting different road object scenarios. Therefore, YOLOv8n is selected as the basic model, which consists of three parts: Backbone, Neck and Head network. The overall structure is shown in Fig 1.

#### 2.1.1 Backbone network.

The backbone network of YOLOv8n adopts a complex convolutional neural network primarily for image feature extraction. It replaces the C3 module with the C2f module based on the YOLOv5 [27] network, making the gradient flow information richer and improving the convergence speed of the model, the C2f structure is shown in Fig 1. Moreover, it is combined with convolutional layers to extract high-level semantic features from the input image [28], further enhancing the network’s feature extraction capability.

#### 2.1.2 Neck network.

The Neck network serves as a crucial link between Backbone and Head networks, comprising the SPPF layer and an improved Path Aggregation Network (PANet) [29]. Its primary function is to fuse the multi-scale features extracted by the Backbone, which is essential for enhancing the overall object detection performance. Specifically, the SPPF layer is used to process features at different scales, which not only improves the feature expression capability, but also speeds up the inference speed of the model compared with SPP. The PANet structure comprises two components: the Feature Pyramid Network (FPN) and the Path Aggregation Network (PAN). FPN adopts top-down method to connect deep and shallow feature maps, while PAN adopts bottom-up feature pyramid network structure, which makes it easier to transfer the bottom information to the top of the upper level, and fully integrates multiscale features.

#### 2.1.3 Head network.

The Head network includes a total of three detection heads corresponding to the feature maps of three different scales in the Neck network, which are mainly responsible for processing the extracted feature maps and generating the final prediction results, including the bounding box coordinates, the object confidence scores, and the category labels. The loss function part uses Binary Cross Entropy Loss (BCE Loss) as the classification loss, and Distribution Focal Loss (DFL) and Complete Intersection over Union (CIoU) [30] Loss as the regression loss. The Head module of YOLOv8 is improved compared with YOLOv5: 1) Improvement of the Coupled-Head structure to Decoupled-Head structure to extract position information and category information respectively; 2) Meanwhile, from the Anchor-Based approach to the Anchor-Free approach, the center point and width-to-height ratio of the object are predicted directly. The above improvements enhance the detection speed and accuracy of the YOLOv8 network model.

## 3 FRCP-YOLO

**Fig 2:**
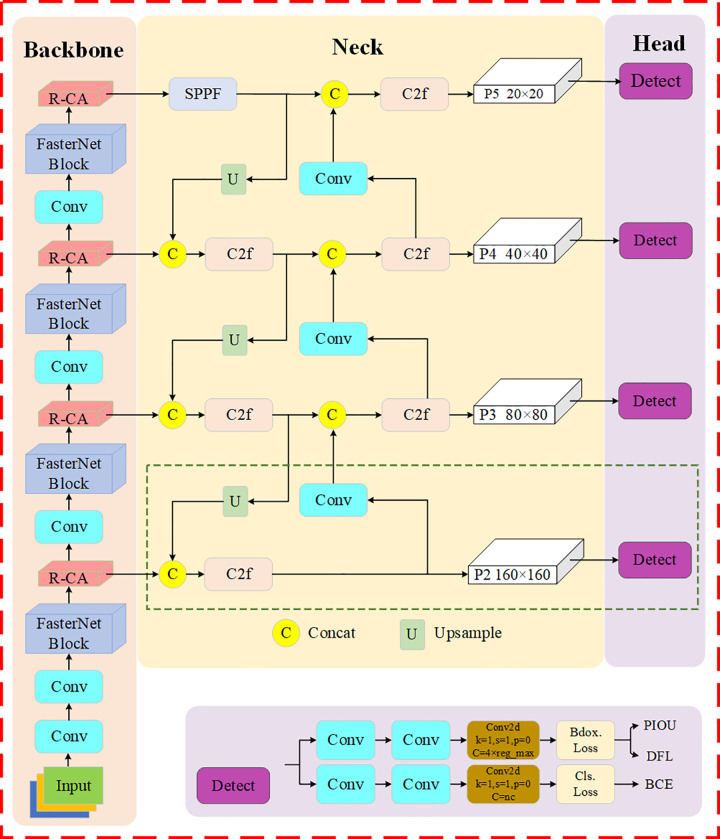
FRCP-YOLO network structure.

Although the YOLOv8n network exhibits satisfactory performance in general object detection tasks, it still suffers from missed and false detections of small objects, insufficient focus capability, and limited detection accuracy in road object detection for autonomous driving scenarios. To address these issues, an improved detection framework named FRCP-YOLO is proposed, as illustrated in Fig 2. The specific improvements over the YOLOv8n network are described in Sections 3.1–3.3.

### 3.1 Improvement of the FRCP-YOLO backbone network

**Fig 3:**
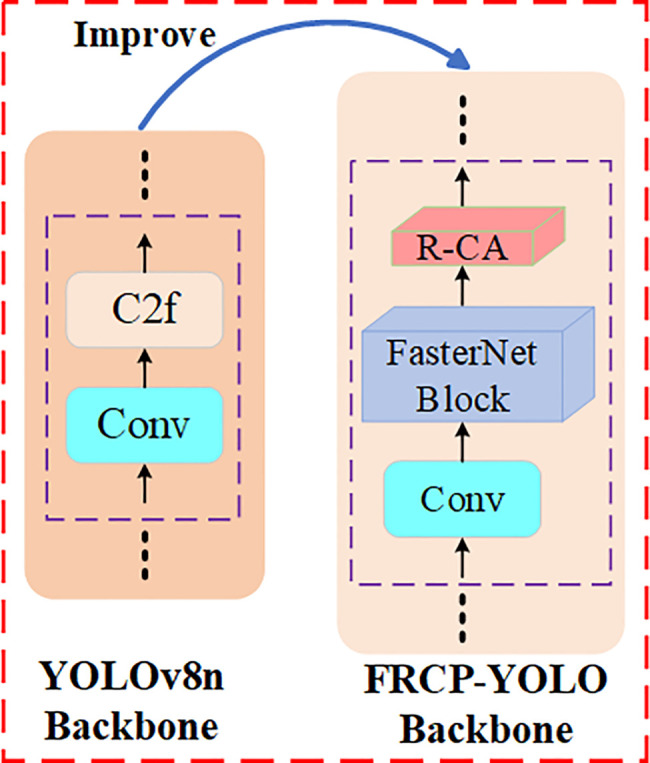
Structural comparison of backbone networks.

This section provides an in-depth analysis of the primary components of the FRCP-YOLO backbone network, including the FasterNet Block and R-CA module, and presents a comparative analysis of the backbone structure prior to and following the improvements, as shown in Fig. 3. FasterNet Block is introduced to reduce the number of model’s parameters and improve computational efficiency. The R-CA module is proposed to address gradient disappearance in model training and enhance the model’s learning ability.

#### 3.1.1 FasterNet block.

**Fig 4:**
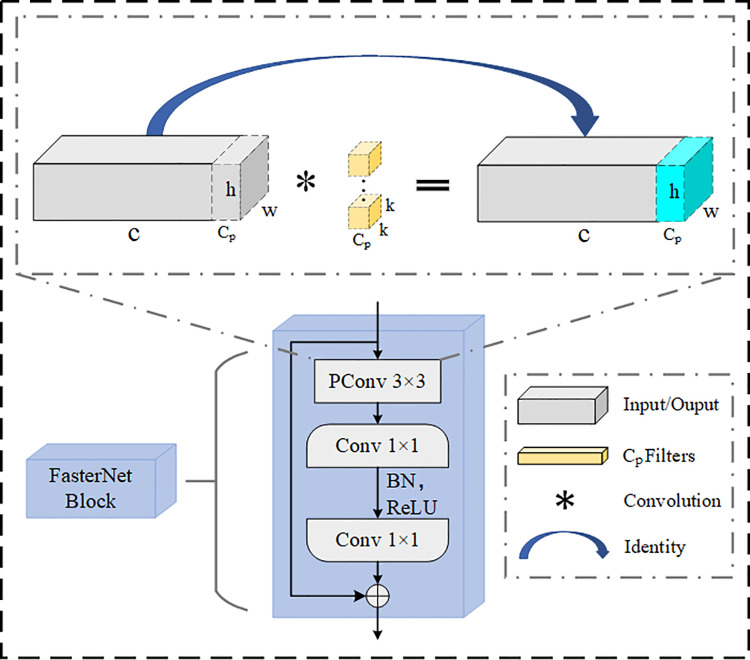
FasterNet Block structure diagram.

The C2f convolution module in the YOLOv8n network significantly improves the model’s feature extraction capability by increasing the number of Bottleneck modules, but also increases the number of parameters. Meanwhile, the feature maps between different channels are highly similar, generating redundant channel information, causing the model to frequently access memory during operation and increase redundant information calculation. To solve the above problems, the FasterNet Block is adopted, which is designed with Partial Convolution (PConv) and Pointwise Convolution (PWConv) as proposed in the FasterNet [31] neural network. The structure is shown in Fig 4.

**Fig 5:**
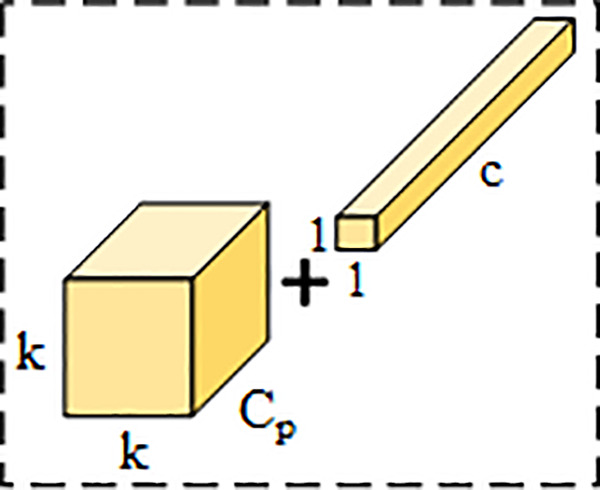
PConv + PWConv illustration.

Lightweight networks such as MobileNets, ShuffleNets, and GhostNet, primarily utilize depthwise convolutions (DWConv) and group convolutions (GConv) for feature extraction. While these methods have achieved notable reductions in model complexity, they often lead to frequent memory accesses and increased computational overhead [31]. In contrast to these conventional lightweight convolutions, PConv applies convolution for feature extraction on only a part of channels $C_{p}$ of the input feature map $I\in {ℜ}^{h\times w\times c}$, and leaving the remaining channels untouched, subtly reduces the computation of redundant feature maps in the channels, largely reduces memory accesses, and improves the detection speed of the model. To more effectively utilize the information of all channels, PWConv is introduced, and the overall receptive field is like a T-shaped convolution, which enhances the model’s attention to the central area of the image. The structure is shown in Fig 5.

Compared with modules such as C2f, FasterNet Block not only solves the problem of frequent memory accesses and channel redundant information calculation of the model, but also reduces model complexity, improves training efficiency. It achieves a better balance between parameter count and detection accuracy, thereby enabling more efficient spatial feature extraction.

#### 3.1.2 R-CA module.

**Fig 6:**
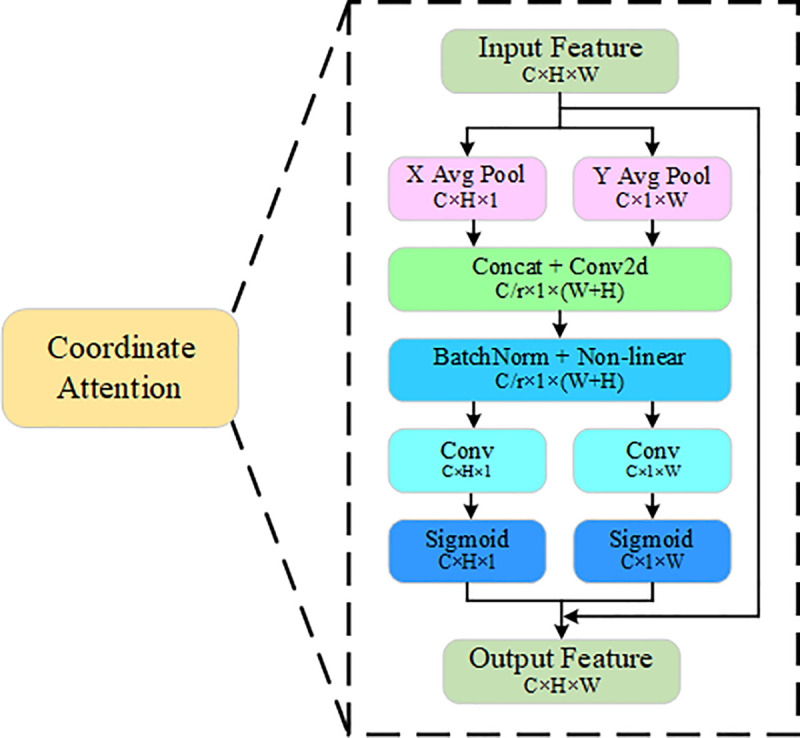
CA structure diagram.

Attention mechanism is one of the common methods to improve the performance of deep neural networks. The widely adopted Squeeze-and-Excitation attention mechanism [32] (SE) reduces computational cost; however, it solely considers channel information and overlooks crucial positional information. In contrast, the CBAM attention mechanism [33] incorporates positional information but is limited to capturing local relationships and involves a large number of parameters. Coordinate Attention (CA) [34] is a lightweight attention mechanism suitable for mobile devices, which enhances accuracy by integrating positional information into channel attention, as illustrated in Fig 6.

Specifically, for any input $X=\left[x_{\text{1}},x_{\text{2}},\cdots ,x_{c}\right]\in {𝔽}^{H\times W\times C}$, where H and W represent the height and width of the input feature map, and the position information is accurately encoded along the horizontal and vertical directions using two pooling kernels of sizes (H, 1) and (1, W) for channel $x_{c}$, respectively, and the attention feature maps in the two directions are obtained. The outputs of the channel $x_{c}$ are shown in Eqs. (1) and (2), respectively:


$$Z_{c}^{h}\left(h\right)=\frac{\text{1}}{W}\sum_{0\leq i\leq W}x_{c}\left(h,i\right)$$ (1)

$$Z_{c}^{w}\left(w\right)=\frac{\text{1}}{H}\sum_{0\leq j\leq H}x_{c}\left(j,w\right)$$ (2)

To fully utilize the obtained information, the above two feature maps are concatenated and convolved to produce an intermediate feature map, as shown in Eq. (3):


$$\begin{array}{c} f=\delta \left(F_{\text{1}}\left(\left[Z^{h},Z^{w}\right]\right)\right) \end{array}$$ (3)

Where [*,*] denotes the concatenation operation along the spatial dimension, δ is the nonlinear activation function, and $F_{\text{1}}$ is the convolutional transformation function. The tensor f is then divided into two separate tensors $f^{w}$ and $f^{w}$ along the spatial dimension, followed by the application of convolutional transformation and nonlinear activation, respectively, as shown in Eqs. (4) and (5):


$$g^{h}=\sigma \left(F_{h}\left(f^{h}\right)\right)$$ (4)

$$g^{w}=\sigma \left(F_{w}\left(f^{w}\right)\right)$$ (5)

Where σ is the sigmoid function, $F_{h}$ and $F_{w}$ perform convolutional transformations on $f^{h}$ and $f^{w}$ respectively, $g^{h}$ and $g^{w}$ are used as the attention weights in the horizontal and vertical directions. The output of the CA mechanism is shown in Eq (6):


$$Y_{CA}=x_{c}\left(i,j\right)\times g_{c}^{h}\left(i\right)\times g_{c}^{w}\left(j\right)$$ (6)

This demonstrates that the CA mechanism captures long-range dependencies along one spatial direction while preserving precise positional information along the other, thereby enhancing the model’s feature extraction capabilities.

**Fig 7:**
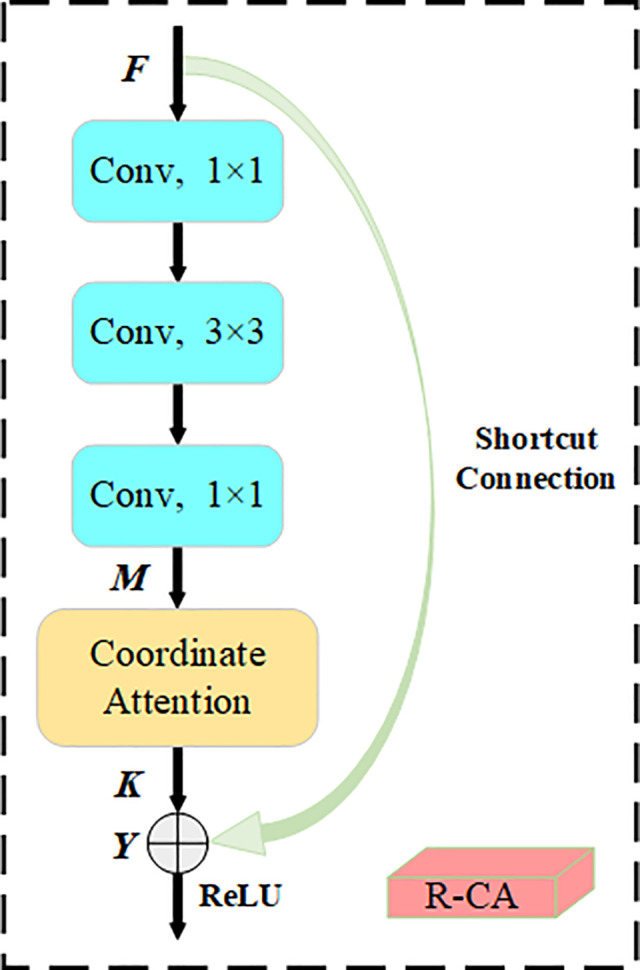
R-CA structure diagram.


As the number of network layers increases, the model encounters issues such as gradient disappearance, which leads to difficult training of the network and reduced model performance. Therefore, the residual block from residual networks [35] is adopted to effectively address the deterioration in model performance caused by an increase in network layers. Combining the CA mechanism and the residual block, the R-CA module is proposed, the structure is shown in Fig 7, and the expressions of R-CA are shown in (9).

$$\begin{array}{c} M=f_{conv}^{1\times 1}\left\{f_{conv}^{3\times 3}\left[f_{conv}^{1\times 1}\left(F\right)\right]\right\} \end{array}$$ (7)

$$K=M_{c}\left(i,j\right)\times g_{c}^{h}\left(i\right)\times g_{c}^{w}\left(j\right)$$ (8)

Y=K+F (9)

Where F represents the input feature map. $f_{conv}^{1\times 1}$ and $f_{conv}^{3\times 3}$ denote standard convolution operations with kernel sizes of 1 × 1 and 3 × 3, respectively. M is the output feature map after standard convolution. K is the feature map processed by the CA mechanism, and Y is the final output feature map.

Incorporating the CA mechanism within the residual block combines the strengths of both modules, enabling the model to focus on critical image regions and enhance its recognition accuracy. The CA mechanism can make the gradient flow propagate better in the model by weighting and adjusting the features. Simultaneously, the residual connection allows the network to effectively capture the difference between input and output, reinforce shallow feature learning, achieve more comprehensive learning of the object features in the image, and enhance the model’s generalization capability.

### 3.2 Improving small object feature extraction network

When YOLOv8 model detects road objects of varying sizes, due to the fact that small objects are easy to be occluded and confused and the feature map area is small, leading to increased chances of missed detection of small objects. This issue is particularly prevalent in complex road scenes containing small objects. To address the above problems and improve the model’s small object detection capability, a dedicated network for small object feature extraction is introduced, as highlighted within the green dotted box in Fig 2. First, the PANet structure in the Neck network is improved by adding an additional branch for small object feature extraction to capture finer details in the image. The number of branches is increased from three to four, resulting in a small object feature map with a high-resolution of 160 × 160 and rich details. Additionally, a detection head is added to the original Head network to process shallow feature maps with more detailed information, further improving the model’s ability to detect small objects.

Aiming at the problems of YOLOv8 model in small object detection, the small object feature extraction network is improved, so that the model more effectively fuses multiscale features, effectively reduces the loss of object features, comprehensively and accurately detects objects of various sizes, and at the same time can cope with the interference of the complex scene such as light change and object occlusion, which improves the robustness of the model.

### 3.3 Improving the bounding box regression loss function

The bounding box regression loss function quantifies the discrepancy between predicted and ground truth bounding boxes, serving as a pivotal component in the object detection algorithm. A lower loss value indicates that predicted box is closer to ground truth box, which results in better detection performance by the model.

**Fig 8:**
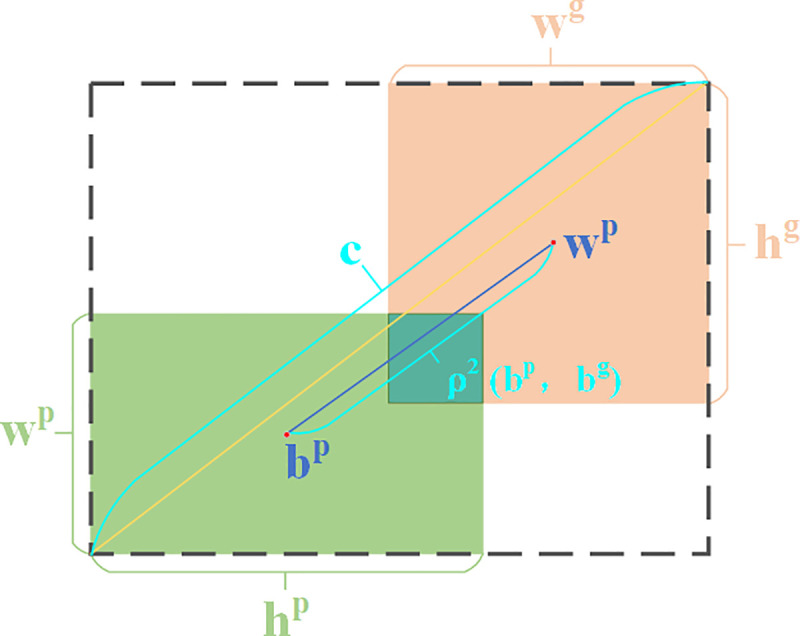
CIoU illustration.


The Complete Intersection over Union (CIoU) [30] bounding box regression loss function is employed in YOLOv8n, which comprehensively accounts for the distance, overlap area, and aspect ratio between bounding boxes. Fig 8 illustrates the intersection between the predicted and ground truth boxes, and Equation (10) present the corresponding calculation formulas.

$$L_{CIoU}=L_{IoU}+\frac{\rho^{\text{2}}\left(b^{p},b^{g}\right)}{c^{\text{2}}}+\frac{\text{4}}{\pi^{\text{2}}}{\left(arctan\frac{w^{g}}{h^{g}}-arctan\frac{w^{p}}{h^{p}}\right)}^{\text{2}}\alpha$$ (10)

$$\alpha =\frac{v}{1-L_{IoU}}$$ (11)

Where $L_{IoU}$ is the intersection loss of the predicted and ground truth boxes, $b^{p}$ and $b^{g}$ denote the centroids of the prediction and ground truth boxes, respectively, c represents the diagonal length of the minimum outer rectangle, and $\rho^{\text{2}}\left(b^{p},b^{g}\right)$ is the distance between the centroids of the two boxes; $w^{p}$, $h^{p}$ and $w^{g}$, $h^{g}$ are the widths and heights of the predicted and ground truth boxes; and v represents the correction factor, while α denotes the weighting coefficients.

**Fig 9:**
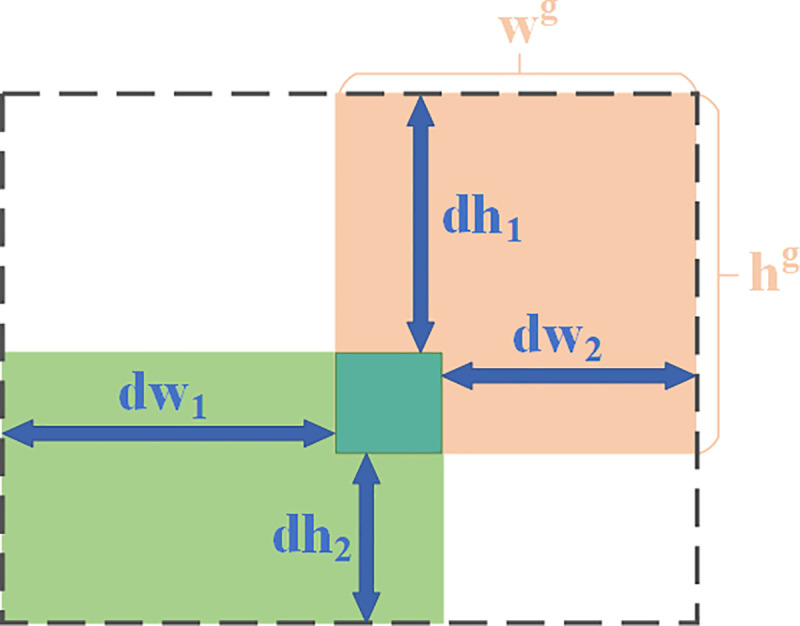
PIoU illustration.


Since the CIoU loss function involves multi-class operations, requires substantial computation time and resources, and exhibits insufficient focus on small objects, it fails to meet the requirements for fast and accurate road object detection in autonomous driving scenarios. Therefore, the efficient Powerful-IoU v2 (PIoU v2) [36] loss function is introduced. The computational formula $L_{PIoU}$ is shown in equation (12), and the intersection schematic between predicted and ground truth boxes is shown in Fig 9.

$$L_{PIoU}=L_{IoU}+1-e^{-p^{\text{2}}}$$ (12)

$$P=\frac{\text{1}}{\text{4}}\left(\frac{d_{w_{\text{1}}}}{w_{g}}+\frac{d_{w_{\text{2}}}}{w_{g}}+\frac{d_{h_{\text{1}}}}{h_{g}}+\frac{d_{h_{\text{2}}}}{h_{g}}\right)$$ (13)

Where P is the penalty factor, $d_{w_{\text{1}}}$ and $d_{w_{\text{2}}}$, $d_{h_{\text{1}}}$ and $d_{h_{\text{2}}}$ represent the absolute distances between the corresponding sides of the predicted and ground truth boxes.

Existing bounding box regression loss functions, such as CIoU, EIoU, and SIoU, suffer from the use of inappropriate penalty factors, which lead to enlarged anchor boxes, slower convergence, and insufficient consideration of object scale [36]. To overcome these limitations, a penalty factor q is incorporated into PIoU v2, which effectively avoids the oversized anchor box and steer the anchor box to return to the ground truth box through a direct and effective way, so that the anchor box more closely match the ground truth box. Additionally, a non-monotonic attention function *u(x)* is introduced to adaptively adjust gradients for the anchor box quality, thereby improving the focus on medium-quality and high-quality anchor boxes, and the formula for the PIoU v2 loss function is shown in the following equation:


$$q=e^{-p},q\in \left[0,1\right]$$ (14)

$$u\left(x\right)=3x\cdot e^{-x^{\text{2}}}$$ (15)

$$L_{PIo\text{U}v2}=u\left(\lambda q\right)\cdot L_{PIoU}$$ (16)

$$L_{PIo\text{U}v2}=3u\left(\lambda q\right)\cdot e^{-{\left(\lambda q\right)}^{\text{2}}}\cdot L_{PIoU}$$ (17)

In this case, the penalty factor p is replaced by q, with the hyperparameter λ set to 1.3. This modification enhances the convergence speed and detection accuracy of the PIoU v2 bounding box regression loss function.

## 4 Experimental results and analysis

### 4.1 Dataset

The autonomous driving dataset KITTI [37], jointly created by the Karlsruhe Institute of Technology in Germany and the Toyota Technological Institute of America, is used in this experiment. It contains a large number of overlapping and small objects, covering a variety of road scenarios such as countryside, city center as well as highway, with a total of Tram, Truck, Car, Person sitting, Pedestrian, Van, Cyclist, Misc, and DontCare 9 categories. After comprehensively considering the actual application of the autonomous driving system, Car, Tram, Truck, and Van are uniformly classified as Car, Person sitting and Pedestrian are uniformly classified as Pedestrian, Cyclist is retained, Misc and DontCare are deleted, and a total of 3 categories (Car, Pedestrian, and Cyclist) are set. Since the test set images in KITTI do not have labeled data, this experiment only uses the labeled training set images, a total of 7481 images, and randomly divides this dataset into the training, validation, and test sets according to the ratio of 8:1:1.

### 4.2 Experimental environment and parameter settings

The platform configuration for this experiment is presented in Table 1. All models were trained for 200 epochs with a batch size of 16 samples. The input image size was set to 640 × 640, the learning rate to 0.01, momentum to 0.937, and the weight decay coefficient to 0.0005.

**Table 1:** Experimental platform configuration.

Configuration	Parameter
CPU	12th Gen Intel(R) Core(TM) i5-12600KF
GPU	NVIDIA GeForce RTX 3060
Operating system	Windows 10
Python	3.8.0
Accelerated environment	CUDA 11.8, CUDNN 8.6.0
Development environment	Pycharm 2023.2.6
Deep learning framework	Pytorch 2.0.0

### 4.3 Evaluation metrics

The present study uses four key evaluation metrics of object detection to comprehensively evaluate the detection performance of different models, including recall (R), precision (P), mean average precision (mAP), and parameters.

The corresponding formula is presented below:


$$P=\frac{TP}{TP+FP}$$

$$R=\frac{TP}{TP+FN}$$

$$mAP=\frac{\text{1}}{N}\sum_{i=1}^{N}AP_{i}$$

Where True positives (TP) represents the number of correctly classified as positive examples, False positives (FP) represents the number of incorrectly classified as positive examples, False negatives (FN) represents the number of incorrectly classified as negative examples. Let $AP_{i}$ denote the average precision of the ith category, and let *N* represent the total number of categories. The number of parameters is used to assess model complexity. As the number of parameters increases, the model becomes more complex and demands greater computational resources.

### 4.4 Experimental results

#### 4.4.1 Ablation experiment.

**Fig 10:**
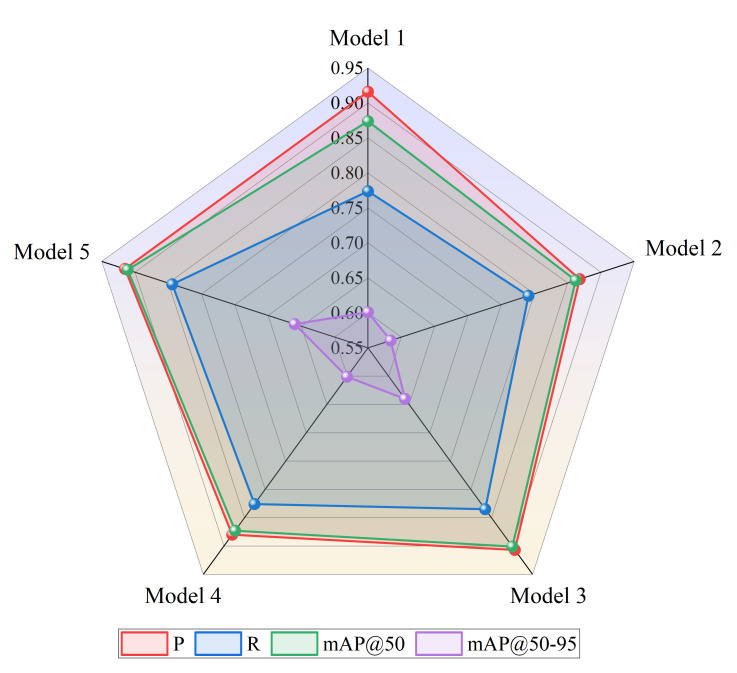
Comparison of ablation experiment results.

To explore the impact of the proposed FasterNet Block, improved small object feature extraction network, and R-CA module on detection performance, the effectiveness of the improvements is verified one by one based on the baseline model. Five groups of ablation experiments are designed based on the KITTI dataset. The test results are shown in Table 2. Fig 10 shows the comparative relationship between the evaluation metrics of different ablation experiments.

**Table 2:** Ablation experiment results.

Model	Method			P	R	mAP@50	mAP@50–95
	A	B	C				
Model 1				**0.916**	0.774	0.874	0.601
Model 2	√			0.868	0.791	0.862	0.584
Model 3	√	√		0.907	0.835	0.901	0.64
Model 4	√		√	0.88	0.826	0.873	0.601
Model 5	√	√	√	0.915	**0.844**	**0.911**	**0.66**

**Note:** A denotes the lightweight and efficient FasterNet Block, B refers to the improved small object feature extraction network, C represents the efficient R-CA module, and model 1 is the YOLOv8n model.

In Model 2, the FasterNet Block is introduced to lighten the original Backbone network, which significantly reduces the complexity of the model, solves the problem of frequent access to memory and channel redundant information, and improves the training efficiency of the model, but the detection accuracy is affected to a certain extent. Model 3 enhances the small object feature extraction network from Model 2, which can be seen in Fig 10 improves all the evaluation metrics, especially R (from 0.791 to 0.835) and mAP@50–95 (from 0.584 to 0.64), which effectively reduces the small object missed detection on the road, and the detection performance has been substantially improved. Compared to Model 2, Model 4 incorporates the R-CA module, resulting in improvements across all evaluation metrics. Specifically, P and R increase by 1.2% and 3.5%, while mAP@50 and mAP@50–95 improve by 1.1% and 1.7%, respectively. These results suggest that the network places greater emphasis on key regions in the image and more effectively learns residual information. Finally, the three proposed improvements are integrated into Model 5. Compared with the original model YOLOv8n, P remains almost unchanged, while R, mAP@50 and mAP@50–95 are increased by 7.0%, 3.7% and 5.9% respectively, which significantly improves the road object detection performance and shows the effectiveness of detecting various object categories.

The ablation experiment results demonstrate that the FasterNet Block, improved small object feature extraction network, and R-CA module proposed in this study effectively improve the performance of the FRCP-YOLO model without any conflict.

#### 4.4.2 Comparative evaluation of bounding box regression loss functions.

Using the Model 5 with better detection performance in section 4.4.1 as the baseline model, experiments were conducted on common loss functions under the same training conditions to study the impact of loss functions on the model’s object detection performance. Table 3 gives the object detection evaluation metrics of the PIoU v2 loss function and other bounding box loss functions on the KITTI dataset.

**Table 3:** Performance comparison of different loss functions.

Loss	P	R	mAP@50	mAP@50–95
CIoU	**0.915**	0.844	0.911	0.66
SIOU	0.912	0.841	**0.924**	0.665
WIoU v1	0.875	0.86	0.915	0.656
WIoU v2	0.91	0.849	0.917	0.663
WIoU v3	0.902	**0.865**	**0.924**	0.661
EIoU	0.909	0.838	0.904	0.645
Focal IoU	0.789	0.682	0.782	0.567
**PIoU v2**	0.906	0.86	**0.924**	**0.667**

As shown in Table 3, the PIoU v2 loss function has better detection performance on Model 5. Compared with the loss function CIoU used by the original network YOLOv8n, R, mAP@50 and mAP@50–95 are increased by 1.6%, 1.3% and 0.7% respectively, and P is slightly reduced. In general, PIoU v2 improves the overall performance of the model better than other loss functions. It not only avoids the anchor box from enlargement, but also enhances the focusing ability of the anchor box. The experimental results show that the PIoU v2 loss function is effective in improving the performance of Model 5.

#### 4.4.3 Comparative experiments of different mainstream algorithms.

**Fig 11:**
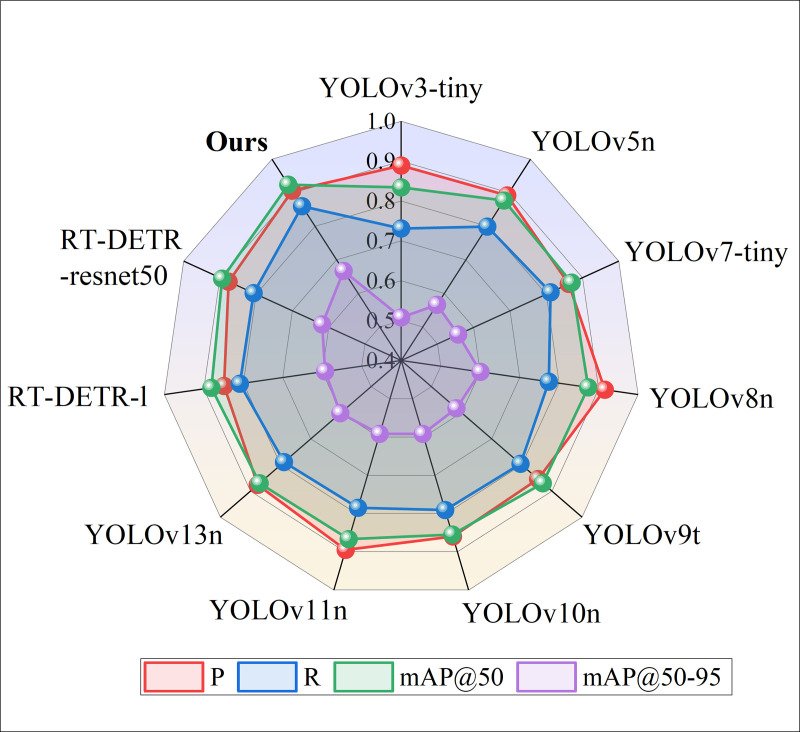
Comparison of detection metrics of different algorithms.

To evaluate the effectiveness of the proposed algorithm in road object detection for autonomous driving scenarios, a comparative experiment was conducted using FRCP-YOLO, YOLOv8, and other mainstream object detection algorithms on the KITTI test set. The results are presented in Table 4 and Fig 11.

**Table 4:** Test indicators of different algorithms.

Algorithm	P	R	mAP@50	mAP@50–95	Parameters/10^6^
YOLOv3-tiny	0.889	0.731	0.834	0.508	8.671
YOLOv5n	0.892	0.799	0.877	0.566	**1.763**
YOLOv7-tiny	0.862	0.812	0.87	0.557	6.013
YOLOv8n	**0.916**	0.774	0.874	0.601	3.006
YOLOv9t	0.854	0.796	0.87	0.583	2.802
YOLOv10n	0.86	0.791	0.855	0.592	2.696
YOLOv11n	0.895	0.785	0.867	0.592	2.583
YOLOv13n	0.876	0.789	0.87	0.602	2.45
RT-DETR-l	0.849	0.809	0.881	0.593	31.99
RT-DETR-resnet50	0.876	0.807	0.894	0.618	41.94
**FRCP-YOLO**	0.906	**0.86**	**0.924**	**0.667**	2.966

Experimental results demonstrate that FRCP-YOLO achieves excellent detection performance while maintaining a lightweight architecture. With only 2.966M parameters, FRCP-YOLO is significantly smaller than larger models such as YOLOv3-tiny (8.671M) and YOLOv7-tiny (6.013M), yet it achieves the highest detection performance among them. Compared with lightweight models such as YOLOv5n, YOLOv9t, YOLOv10n, YOLOv11n, and YOLOv13n, FRCP-YOLO delivers superior detection accuracy while maintaining a compact design. Furthermore, compared with Transformer-based object detectors such as RT-DETR-l and RT-DETR-ResNet50, FRCP-YOLO demonstrates a clear advantage by achieving a substantial reduction in parameter count and computational cost while sustaining leading detection accuracy. Relative to the baseline YOLOv8n model, FRCP-YOLO reduces the number of parameters by 4% while significantly enhancing detection performance, with R increased by 8.6%, and mAP@50 and mAP@50–95 improved by 5.0% and 6.6%, respectively. Overall, these results clearly demonstrate that FRCP-YOLO effectively combines a lightweight design with robust and accurate detection capability for road object detection tasks.

### 4.5 Verification of the generalization ability of the FRCP-YOLO model

**Fig 12:**
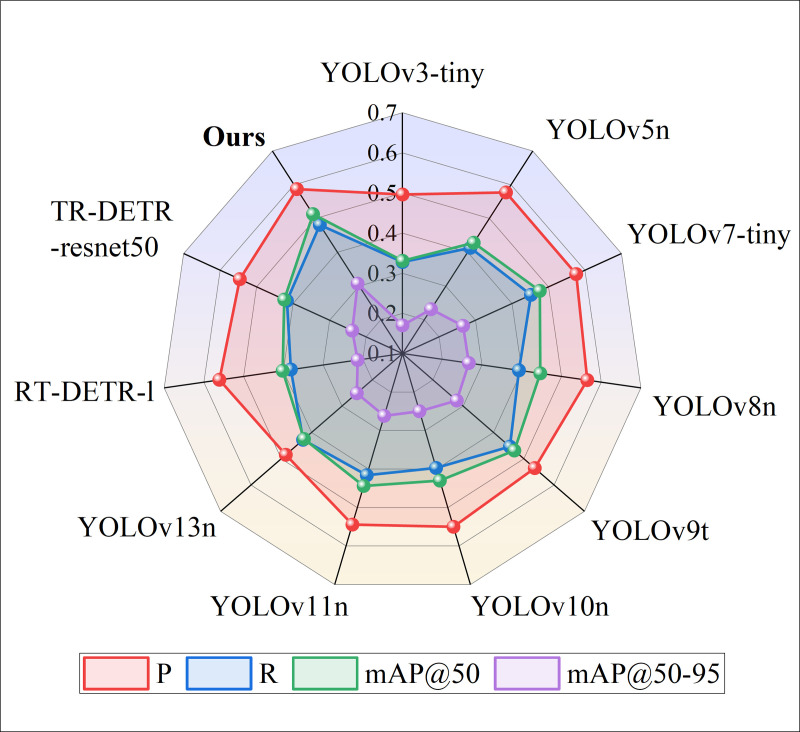
Comparison of detection metrics of different algorithms.

To further verify the generalization ability of FRCP-YOLO, road detection experiments were conducted on the BDD100K [38] dataset released by the Berkeley AI Lab (BAIR) in 2018 under various weather conditions (such as sunny, cloudy, rainy, etc.) and across different times of day and night. Considering that the number of images containing Motor and Bike objects in the BDD100K dataset is small and the characteristics of the two are similar, the two are classified as Two-Wheelers, and then Pedestrian, Car, Truck, Bus, and Two-Wheelers are used as the objects of road object detection. A random selection of 10,000 images from the BDD100K dataset is used to evaluate the model’s generalization ability, which is divided into the training set, validation set, and test set according to the ratio of 8:1:1. The performance of FRCP-YOLO was compared with other mainstream algorithms from Section 4.5 on this dataset for object detection, with the experimental results presented in Table 5 and Fig 12.

**Table 5:** Test indicators of different algorithms.

Algorithm	P	R	mAP@50	mAP@50–95
YOLOv3-tiny	0.496	0.328	0.331	0.17
YOLOv5n	0.577	0.413	0.428	0.231
YOLOv7-tiny	0.576	0.452	0.476	0.266
YOLOv8n	0.565	0.393	0.447	0.267
YOLOv9t	0.536	0.454	0.469	0.279
YOLOv10n	0.55	0.397	0.43	0.25
YOLOv11n	0.544	0.416	0.444	0.262
YOLOv13n	0.485	0.428	0.425	0.251
RT-DETR-l	0.561	0.381	0.402	0.213
RT-DETR-resnet50	0.546	0.417	0.424	0.238
**FRCP-YOLO**	**0.587**	**0.481**	**0.513**	**0.307**

The experimental results show that the proposed road object detection model outperforms other network models across all metrics. Compared with the baseline model YOLOv8n, FRCP-YOLO has higher detection performance. Specifically, P increased by 2.2%, R increased by 8.8%, mAP@50 and mAP@50–95 increased by 6.6% and 4%, respectively. A large number of comparative experimental results show that the FRCP-YOLO can accurately perceive the external environment in complex road scenes and realize accurate detection of road objects, with strong adaptability and generalization ability.

## 5 Conclusion

Aiming at the problems of small object missed detection, large number of parameters, and low detection accuracy of existing models in complex road scenes, a lightweight and efficient road object detection model named FRCP-YOLO is proposed. Specifically, the lightweight and fast FasterNet Block is utilized to extract features from a part of the input channels, by cutting down model’s parameters and redundant computation; combined with the Coordinate Attention mechanism and residual block, an efficient R-CA module is proposed to enhance the detection of objects of interest and overcome the problems of gradient disappearance and performance degradation; the small object feature extraction network is improved to capture tiny details in the image, reduce the loss of object information, decrease the impact of complex road scenes, and improve the robustness of the model; PIoU v2 is introduced as the bounding box loss function to guides anchor boxes to regress along efficient paths, resulting in faster convergence and improved detection accuracy.

Experimental results show that FRCP-YOLO performs better in road object detection tasks based on the KITTI dataset and the BDD100K dataset containing complex road environments. Compared with the baseline model, its accuracy increased by 5.0% and 4% respectively (in terms of mAP@50), while R increased by 8.6% and 8.8%, and the number of parameters decreased by 4%. Compared with the other mainstream algorithms, FRCP-YOLO has achieved detection accuracy superior to the other mainstream algorithms while realizing lightweight, has better generalization, effectively solves the problems of small object missed detection, large number of parameters and low detection accuracy in complex road scenarios, and is suitable for object detection of autonomous driving vehicle in complex roads. This study evaluates the improved model from multiple dimensions such as detection accuracy and the number of parameters, and provides new ideas and methods for subsequent autonomous driving visual perception research. Despite these promising results, the present study still has certain limitations, and future work will focus on optimizing the model from the following two aspects:

(1) In the experiment, only major road objects such as pedestrians and motor vehicles were detected. In the future, road objects such as traffic lights and traffic signs will be included in the research scope.
(2) The next step will be to consider adding a semantic segmentation network based on the FRCP-YOLO model to complete lane line detection and realize the joint detection of road objects and lane lines for autonomous driving vehicles.
